# Isoniazid-historical development, metabolism associated toxicity and a perspective on its pharmacological improvement

**DOI:** 10.3389/fphar.2024.1441147

**Published:** 2024-09-19

**Authors:** Jishnu Sankar, Anjali Chauhan, Ramandeep Singh, Dinesh Mahajan

**Affiliations:** ^1^ Centre for Drug Discovery, BRIC-Translational Health Science and Technology Institute, Faridabad, Haryana, India; ^2^ Department of Pharmaceutical Chemistry, Delhi Institute of Pharmaceutical Sciences and Research, New Delhi, India; ^3^ Centre for Tuberculosis Research, BRIC-Translational Health Science and Technology Institute, Faridabad, Haryana, India

**Keywords:** isoniazid, metabolism, hepatotoxicity, peripheral neuropathy, N-acetyltransferase, prodrug

## Abstract

Despite the extraordinary anti-tubercular activity of isoniazid (INH), the drug-induced hepatotoxicity and peripheral neuropathy pose a significant challenge to its wider clinical use. The primary cause of INH-induced hepatotoxicity is *in vivo* metabolism involving biotransformation on its terminal -NH_2_ group owing to its high nucleophilic nature. The human *N-acetyltransferase-2* enzyme (NAT-2) exploits the reactivity of INH’s terminal -NH_2_ functional group and inactivates it by transferring the acetyl group, which subsequently converts to toxic metabolites. This -NH_2_ group also tends to react with vital endogenous molecules such as pyridoxine, leading to their deficiency, a major cause of peripheral neuropathy. The elevation of liver functional markers is observed in 10%–20% of subjects on INH treatment. INH-induced risk of fatal hepatitis is about 0.05%–1%. The incidence of peripheral neuropathy is 2%–6.5%. In this review, we discuss the genesis and historical development of INH, and different reported mechanisms of action of INH. This is followed by a brief review of various clinical trials in chronological order, highlighting treatment-associated adverse events and their occurrence rates, including details such as geographical location, number of subjects, dosing concentration, and regimen used in these clinical studies. Further, we elaborated on various known metabolic transformations highlighting the involvement of the terminal -NH_2_ group of INH and corresponding host enzymes, the structure of different metabolites/conjugates, and their association with hepatotoxicity or neuritis. Post this deliberation, we propose a hydrolysable chemical derivatives-based approach as a way forward to restrict this metabolism.

## Graphical Abstract

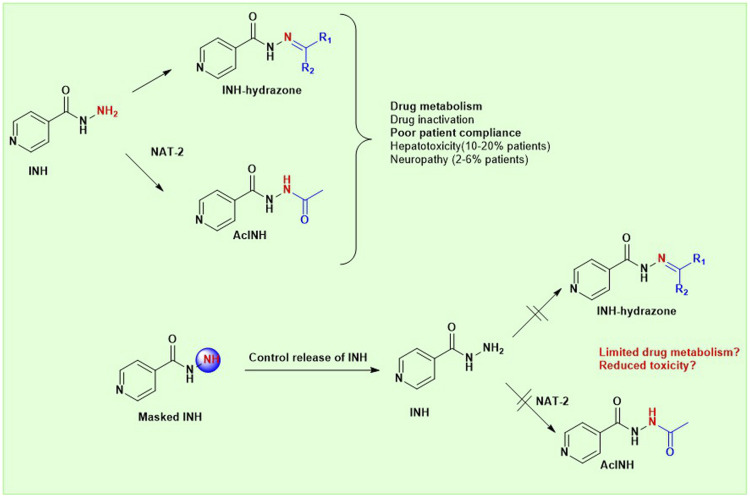

## 1 Introduction

Tuberculosis (TB) is an infectious disease caused by *Mycobacterium tuberculosis* (*Mtb*), with a high economic burden and a considerable mortality risk. There is documented evidence in the literature suggesting efforts on anti-tubercular drug discovery dated back to 1930 (Wells, 1932). Streptomycin (STM) was the first antibiotic that demonstrated a robust therapeutic effect against TB. This was followed by the discovery of the bacteriostatic potential of *p*-aminosalicylic acid (PAS) (Lehmann, 1964; Fitzgerald and Bernheim, 1948). This led to a study that evaluated the combination of STM and PAS in randomised clinical trials against *Mtb* infection (Crofton et al., 1952). Later, the discovery of isoniazid (INH) initiated a combined triple therapy evaluation of INH, STM and PAS. This study proved the efficacy of this combination involving a treatment of 24 months (Capon, 1954). Further research to develop a therapeutic regimen aiming for a shorter treatment period resulted in the discovery of ethambutol (ETA) (Shepherd et al., 1966; Thomas et al., 1961) and pyrazinamide (PZA) (Malone et al., 1952). Later, the combination of first-line drugs like INH, RIF, PZA, and ETA resulted in a modern-day therapeutic regimen of 6 months with enhanced efficacy (Society, 1984). The emergence of drug-resistant *Mtb* strains against INH and/or RIF and drug-associated toxicity put forth a significant challenge in treatment (Günther, 2014). Bedaquilline, pretomanid and delamanid are newly approved drugs and are included in the treatment regimen of multidrug-resistant tuberculosis (MDR-TB) by WHO (Organization, 2014; Pym et al., 2016). Some other drugs (such as carbapenems and linezolid) are also repositioned for the treatment of individuals with multidrug-resistant/extensively drug-resistant tuberculosis (MDR/XDR-TB) (Table 1) (Tiberi et al., 2016a; Tiberi et al., 2016b; Sotgiu et al., 2015).

**TABLE 1:** The treatment regimen for TB patients (Bagcchi, 2023).

Target population	Duration	Regimen	
		Initial phase	Continuation phase
Overall TB Patients	6 months (2 + 4)	2 months of INH, RIF, PZA and ETA	4 months of INH and RIF
Children and adolescents (age: 3 months to 16 years)	4 months	2 months of INH, RIF and PZA, sometimes ETA	2 months of INH, RIF
Patients of age 12 years	4 months	4 months of INH, RPT, PZA and MFX	
Group of medicines being used for MDR-TB regimens (World Health Organization, 2022)			
Group AInclude all three medicines		Levofloxacin or MoxifloxacinBedaquilineLinezolid	
Group BAdd one or both medicines		Clofazimine Cycloserine or terzidone	
Group CAdd to complete the regimen and when medicines from Groups A and B cannot be used		EthambutolDelamanidPyrazinamideImipenem-cilastatin or meropenemAmikacin or streptomyxinEthionamide prothionamide P-aminosalicylic acid	

INH is an important drug and is being prescribed globally as a key component of TB treatment including individuals with latent tuberculosis infection (LTBI), particularly in high-burden TB regions. Despite being one of the most successful anti-TB drugs, the history of development and clinical limitations of INH and possible approaches to address this are not comprehensively reviewed. The first section of this review focuses on the genesis and historical development of INH along with various known (validated as well as non-validated) mechanisms of action contributing to the overall anti-tubercular activity of INH. Post this section, we summarise the different clinical trials in chronological order, highlighting the treatment-associated toxicity and their occurrence rate. This section follows a detailed discussion of literature reports encapsulating various *in vivo* metabolic chemical transformations, the formation of various INH metabolites/conjugates, and their association with hepatotoxicity or neuritis. Post this deliberation, we propose that a hydrolysable chemical derivative-based approach by masking the terminal -NH_2_ group of INH can potentially be a way forward to restrict this *in vivo* metabolism and associated toxicities.

## 2 Discovery, development and mechanism of action of INH

**FIGURE 1:**
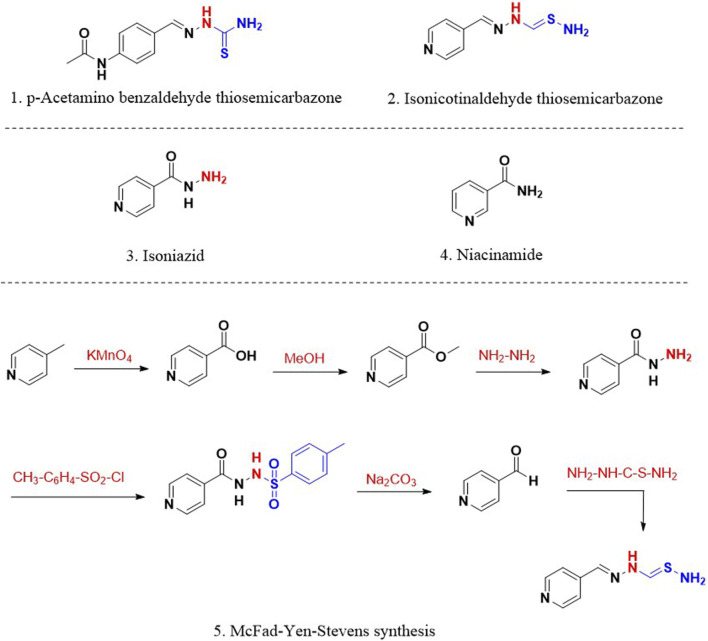
1. p-Acetaminobenzaldehyde thiosemicarbazone 2. Isonicotinaldehyde thiosemicarbazone; 3. Isoniazid (INH); 4. Niacinamide; 5. McFad-Yen-Stevens synthesis of thiosemicarbazone.

Following the discovery and clinical use of STM and PAS, the focus was shifted to evaluating *p*-acetamino benzaldehyde thiosemicarbazone (1; Figure 1) for its antitubercular activity. Though the compound 1 was found active in clinical trials, the drug had specific side effects that denied its therapeutic establishment. Based on the existing knowledge around thiosemicarbazones, Yale et al. (Kauffman, 1978; Yale et al., 1953) designed and synthesised isonicotinaldehyde thiosemicarbazone (2; Figure 1) using a six-step (5; Figure 1) McFad-Yen-Stevens sequence of synthesis (McFadyen and Stevens, 1936). The intended research work around this molecule led to serendipitous discovery and development of INH. During the synthesis of isonicotinaldehyde thiosemicarbazone (Lehmann, 1964), a chance observation that INH is a bioactive intermediate led to its fortuitous discovery and significant antitubercular activity. Chemically, INH is an amide derivative of 4-pyridinecarboxylic acid (3; Figure 1) known as isonicotinic acid. Concurrently, encouraged by the discovery of antitubercular activity of niacinamide (4; Figure 1) an amide derivative of 3-pyridinecarboxylic acid by Chorine (Chorine, 1945) and Huant (Huant, 1945), Fox and his fellows (Fox, 1952) studied pyridinecarboxylic acids and their derivatives for antitubercular activity. This work also established the antitubercular potential of INH (Kauffman, 1978). The newly found drug INH also demonstrated activity against strains resistant to STM and PAS (Long, 1958) and reduced the number of patients who were intolerant to STM and PAS. Anorexia and gastrointestinal disturbances were the significant drawbacks of PAS. In addition, INH reported enhanced activity in patients with tuberculous laryngitis compared to STM (Lumsden and Swoboda, 1952). Certain patients under INH treatment developed mild side effects such as insomnia, irritability, dizziness and drowsiness. However, considering the extraordinary efficacy of INH, these mild side effects were disregarded during that time. Unfortunately, the clinical trials with a treatment period of more than 12 weeks accounted for the emergence of drug-resistant strains against INH (Middlebrook, 1954).

**FIGURE 2:**
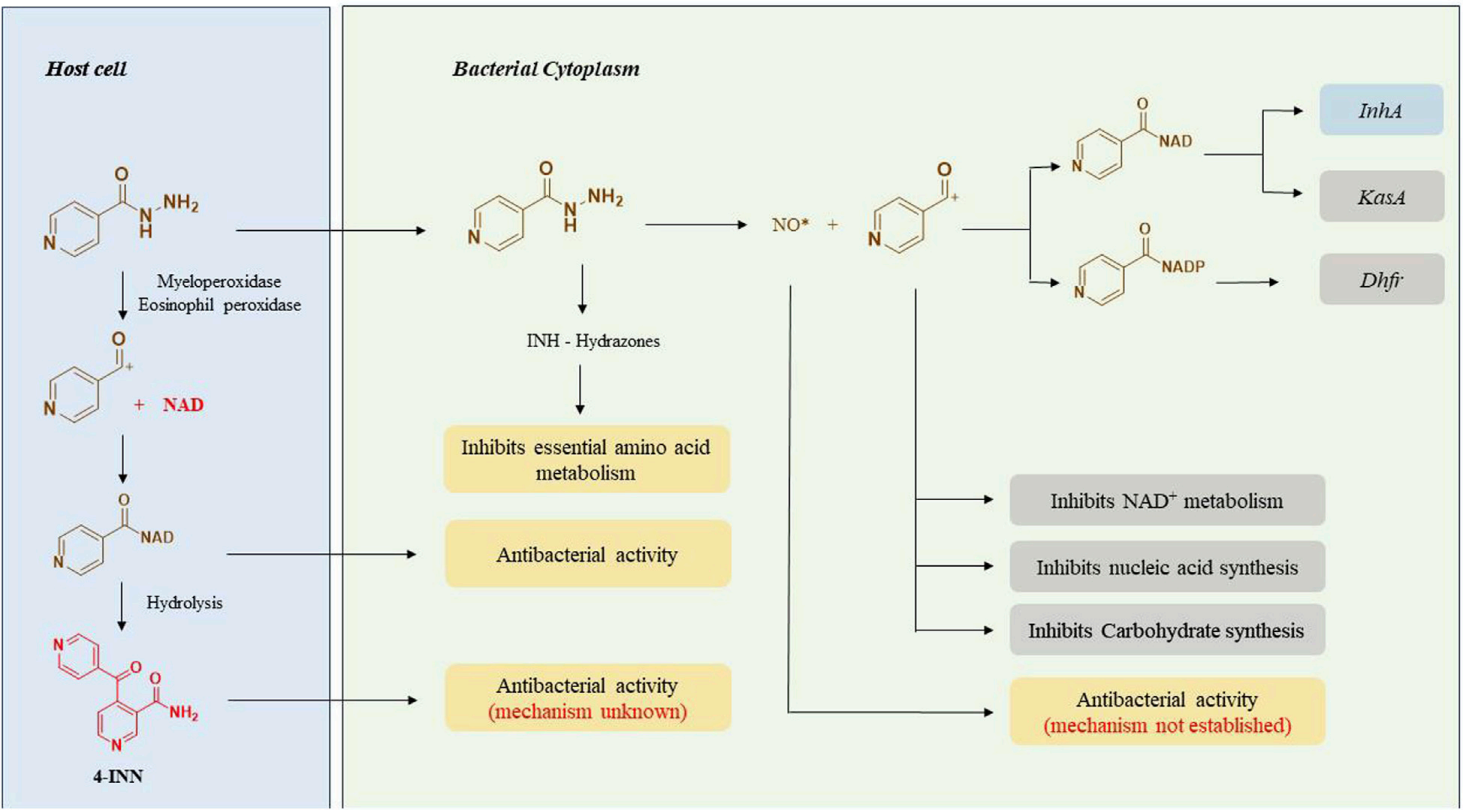
A pictorial overview on the mechanism of action of INH.

The capability of INH to cross the cell membranes simply by passive diffusion and selective action against *Mtb* accords with its high therapeutic potential (Bardou et al., 1998). INH is a unique prodrug that gets activated inside the *Mycobacterium* by the catalase-peroxidase enzyme (KatG) of *Mtb* (Johnsson and Schultz, 1994; Winder, 1960). INH itself is nontoxic to the bacteria whereas its intrabacterial activation by bacterial KatG enzyme releases reactive free radical species that interact with various cellular components of bacteria such as nucleic acids, proteins and lipids (Figure 2). These free radicals directly or as their adducts contribute to the overall antitubercular activity of INH. The activation process of INH is suspected to depend upon intracellular Mn^2+^ concentration, oxidants like superoxide, hydrogen peroxides and alkyl hydroperoxides (Rozwarski et al., 1998; Davidson and Takayama, 1979; Argyrou et al., 2006a). A series of carbon, nitrogen, and oxygen free radicals are generated upon activation of INH by the action of bacterial enzymes. Acyl radicals, acyl peroxy radicals, pyridyl radicals, and isonicotinoyl radicals are formed due to KatG-mediated activation of INH. Among these, the isonicotinoyl radical is the most active and well-studied species as far as the mechanism of action of INH is concerned. Isonicotinoyal radical kills the bacteria by forming an adduct with nicotinamide adenine dinucleotide (NAD^+^) (Rozwarski et al., 1998). This adduct binds with InhA, an enoyl acyl carrier protein reductase enzyme that inhibits its activity. This enzymatic inhibition leads to the restricted synthesis of mycolic acid, a critical component required for the integrity of bacterial cell wall (Davidson and Takayama, 1979). Extensive studies on INH-NAD adduct conclude that the *S* isomer of the adduct is responsible for the active inhibition of InhA (Rozwarski et al., 1998; Argyrou et al., 2006a; Timmins and Deretic, 2006) whereas the *R* isomer of the adduct is proposed as a dihydrofolate reductase (DHFR) inhibitor based on the studies on its co-crystallisation with INH (Argyrou et al., 2006b). However, this finding was challenged and opposed later since INH-DHFR interaction was unproven with detailed *in-vivo* experiments (Wang et al., 2010). DHFR plays a critical role in nucleic acid synthesis in *Mtb,* but the inhibition of DHFR by INH is still debatable (Timmins and Deretic, 2006; Miesel et al., 1998). KasA, a beta-keto acyl carrier protein synthase, has been reported as an additional target of INH based on protein profiling studies of INH-treated *Mtb*. This argument was supported by the formation of an 80 KDa complex of INH, KasA, and meromycolate extension acyl carrier protein (AcpM) and the accumulation of saturated hexacosanoic acid, a precursor of mycolic acid synthesis. AcpM is a small protein that carries the fatty acyl chains across the FAS-II system during mycolic acid synthesis. Hence, it was proposed that INH restricts fatty acid elongation by inhibiting KasA (Mdluli et al., 1998). In addition to these three modes of action of INH, there are some other proposed contributory mechanisms that need further detailed evaluations. In the early 1960s, INH activity was considered to be associated with the inhibition of nucleic acid synthesis (Gangadharam et al., 1963), glycolysis and carbohydrate synthesis (Winder and Rooney, 1970) and NAD^+^ metabolism inside the bacteria (Winder and Collins, 1968; Herman and Weber, 1980). The identification of a novel metabolite, 4-isonicotinoylnicotinamide (4-INN), using LCMS analysis of urine samples of TB patients was a significant finding. The presence of this metabolite in culture-negative patients and uninfected mice treated with INH led to a conclusion that 4-INN is derived by the hydrolysis of INH-NAD^+^ adduct produced in host cells. It was proposed that 4-INN could implement potential antibacterial activity (Mahapatra et al., 2012) due to its structural similarity with truncated INH-NAD^+^ adducts (Delaine et al., 2010). Concurrently, metabolomic studies done in urine samples from TB patients characterised five novel hydrazones formed by the reaction of INH with various endogenous keto acids (Li et al., 2011). It is proposed that these hydrazones could exert an antitubercular activity by acting as an intermediate in the amino acid metabolism of *Mycobacterium* (Du Preez and Loots, 2018). The formation of NO* radical from the Hz chain of INH during KatG activation has been observed using N^15^ labelled INH. This radical is also suspected to inhibit bacterial growth (Timmins et al., 2004). INH is also proposed to modulate the host immune system (Khan et al., 2016). The human immune system gets activated as soon as the *Mtb* infects the lungs. As a first line of defence, the macrophages try to kill the bacteria through phagocytosis. Sometimes, macrophages fail to inhibit the infection completely. In that case, it just assembles around the bacteria to form granuloma and facilitates the stay of bacteria in a dormant stage, called the latent stage of TB infection (Guirado and Schlesinger, 2013). During this dormant stage, host immune bodies create a high oxidative stress in which bacteria remain viable (Voskuil et al., 2011) however macrophages undergo necrosis (Dallenga et al., 2017) beyond a threshold level. Recent reports point out that INH can improve this tolerance level of host immune bodies and prevent their oxidative necrotic death. Thus, INH indirectly inhibits the growth of the bacterium in its latent stage (Khan et al., 2016; Khan SR. et al., 2019). In addition to intrabacterial activation of KatG, INH is also activated by neutrophil myeloperoxidase (Khan et al., 2016) and eosinophil peroxidase (Babu et al., 2019) in the host body that ultimately results in INH-NAD adduct which further transits to the bacterial cytoplasm and exhibits antitubercular activity by inhibiting its cell wall synthesis.

## 3 Clinical studies of INH (efficacy vs safety)

This section provides a detailed analysis of various clinical studies highlighting the adverse effects associated with INH treatment. It summarizes different global clinical trials involving different doses of INH, geographical locations, number of subjects, and treatment regimens, along with reported adverse events.

**FIGURE 3:**
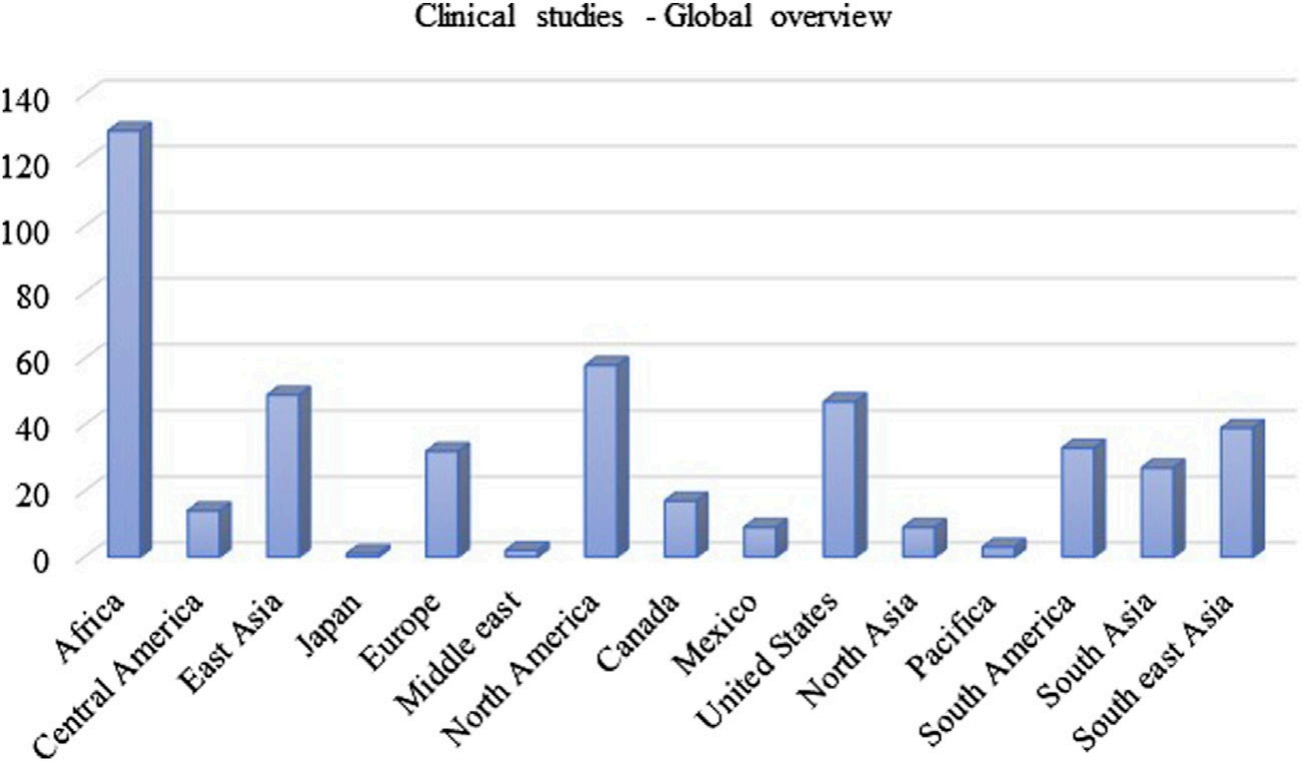
The number of clinical trials done using INH treatment in different countries (as per data available till 15th February 2024; Source: https://ClinicalTrials.gov).

Immense research and clinical trials have been performed to study the effectiveness, safety and pharmacology of INH since its discovery. Figure 3 summarises the number of clinical trials performed globally using INH till February 2024. INH treatment is reported to induce elevated liver functional markers in 10%–20% of the subjects globally. INH-induced risk of fatal hepatitis is about 0.05%–1%. The incidence of peripheral neuropathy is estimated to be between 2%–6.5% with a higher percentage in the elderly population (Badrinath and John, 2018). Under-reporting of adverse incidents associated with INH therapy is common even in Western countries with established healthcare infrastructure (Hayashi et al., 2015). Although the incident rate of adverse events like hepatotoxicity and neuropathy seems to be low, it actually translates to a very high absolute number of affected patients considering the high overall global prevalence of TB infection. Initial studies with a three-month regimen of INH provided a strong sign of efficacy against TB infection. Rapid offset of fever, reduced sputum and cough were the key observed benefits of INH treatment in those studies (Robitzek and Selikoff, 1952). Also, INH treatment significantly improved chronic respiratory insufficiency and rapid healing of tuberculosis laryngitis. However, minor side effects such as insomnia, fainting, dizziness, and irritability were also observed (Crofton et al., 1952; Lumsden and Swoboda, 1952). Adminstration of a higher dose of INH results in grand mal seizures as a result of photic stimulation since hydrazide compounds are capable of hampering normal cerebral cortical functions (Bhide et al., 1978). Later, scientists and physicians invested efforts to understand the toxicity profile of INH through specifically designed clinical trials. The randomised clinical trials conducted by the British Medical Research Council (BMRC) in 1953 found a significant increase in drug resistance cases during the standalone administration of INH (Crofton et al., 1952). This study led to a debate on the administration of INH as a standalone drug or in combination with PAS/STM, for better clinical management and a favourable outcome. Usual gastric disturbances and selective intolerance developed by PAS or STM raised a challenge for physicians to continue with an altered regimen of INH in combination. However, detailed clinical studies on patients of different ages with acute and chronic pulmonary TB using INH-STM, STM-PAS and INH alone proved that the INH-STM combination is a safer therapeutic regimen. Lower blood sedimentation rate (BSR), rapid lowering in body temperature with improvement in the health of patients were the remarkable features of this new treatment regimen. Further minor side effects like constipation, postural hypotension, nocturia, increased dyspnoea and paraesthesia were reported with INH treatment. Apart from these minor side effects, the occurrence of adverse effects like psychoses, convulsions, and severe allergic reactions during INH therapy were also found to be of concern. Careful clinical observations also suggested the appearance of INH resistance (40%–80% of patients) within 16 weeks of treatment (Coates et al., 1954; Hutton et al., 1956). Between all these clinical observations, it was also speculated that INH could potentially induce tumours in TB patients since similar results have been obtained in animals treated with INH (Biancifiori and Severi, 1966; Bhide et al., 1978). However, vigilant and comprehensive clinical analysis disregarded this speculation (Ferebee and Comstock, 1967; Clemmesen and Hjalgrim-Jensen, 1979). Various clinical studies were carried out between the early 1960 to late 1970 to determine the possible risk factors or adverse effects vis-à-vis the clinical benefits of INH treatment. In parallel, an *ad hoc* committee of the American Thoracic Society (ATS) approved the use of INH in chemoprophylaxis as a preventive treatment of TB in 1965. In their report, ATS stated, *“The extensive trials conducted by the United States Public Health Service show a consistent reduction of morbidity in treated groups, it seems reasonable to expect that chemoprophylaxis can reduce the future morbidity from TB, in high-risk groups by some 50 to 75 percent. The extensive use of chemoprophylaxis would likely reduce by 300,000 the total number of cases in the United States in the next 15 years”.* The committee suggested a chemoprophylaxis regimen of a single dose of 300 mg daily, for adults and 10 mg/kg for children for 12 months. All the individuals who tested positive for the tuberculin test were considered under this preventive treatment (Society, 1967). It appears that the initial fascinating outcome on the clinical efficacy of INH overwhelmed the reported associated adverse events. In 1969, Assem et al. (1969) revealed that INH treatment may cause jaundice and liver damage. These studies highlighted hepatitis and jaundice as two frequently reported adverse effects of INH treatment. Subsequently, liver damage and hepatic cell necrosis were also reported as common adverse effects among patients on INH therapy. A case study (1965–1969) in the United States revealed definitive proof of hepatotoxicity associated with INH therapy. This study involved a pool of 167 patients who were on antitubercular therapy, including both *Mtb*-infected and non-infected patients (Scharer and Smith, 1969). Around 10% of patients expressed an uncontrolled elevation of Serum Glutamic Oxalate Acetic Transaminase (SGOT) and Serum Glutamic Pyruvic Transaminase (SGPT) levels within 2 months of therapy. Liver biopsies of patients determined hepatocellular damage. Before this study, Doppelt and Hensler also observed elevated levels of transaminase enzymes in patients treated with INH (Doppelt and Hensler, 1962). These studies provided conclusive evidence that standalone administration of INH is responsible for serious hepatic abnormalities; hence, the dose of chemoprophylactic treatment of INH should be reconsidered. In the same year, the Centre for Disease Control and Prevention (CDC) called up a conference to evaluate the risks of INH prophylaxis and the use of INH. This conference officially documented that *“INH associated liver disease is viewed as an unpredictable hypersensitivity response”*. Following the recommendations of the CDC, the United States Public Health Service conducted a clinical study on 36,000 volunteers to evaluate the toxic reaction of INH. Out of this, 1.9% of household contacts and 6.6% of patients reported toxic reactions to INH therapy. It is important to highlight that children are resistant to adverse reactions induced by INH compared to adults, and this study included a significant number of pediatric subjects. This made the study outcome biased (Ferebee, 1970). In 1974, the CDC revised its guidelines and also suggested to study other combination regimens in order to identify a therapy having a better safety profile. This thought process and subsequent efforts led to the development of a modern-day combination regimen involving RIF, PZA, ETA, and INH with a reduced rate of hepatoxicity and patient compliance. A summary of these clinical studies is mentioned in Supplementary Table S1. The summarized data in Supplementary Table S1 highlights the small but significant cases of hepatotoxicity with newly attempted combination therapies of INH. Hepatitis and jaundice have been reported as the prominent side effects in most of the clinical studies. Along with hepatotoxicity, INH induced peripheral neuropathy (PNP) is another major issue in clinics. The “burning feet sensation” of PNP was the major feedback in initial reports of adverse drug reactions of INH (Jones and Jones, 1953). PNP is associated with deficiency of vitamin B_6_. INH reacts with Vitamin B_6_ non-enzymatically to form corresponding hydrazones, leading to the depletion of this vital nutrient. PNP is a dose dependent toxic effect of INH. Toxicity studies reported from higher dose administration of INH to 119 TB patients accounted for 44% peripheral neuritis and about 10% gastric intolerance (Biehl and Nimitz, 1954). A clinical study of INH with a dose of 20 mg/kg reported neuritis among 40% of the total patient population. Within five to 7 weeks of treatment, all the patients reported burning feet sensation, muscle weakness and numbness (Biehl and Vilter, 1954a). A study conducted by the Tuberculosis Chemotherapy Centre (TCC, India) on 338 malnourished TB patients involving INH doses of 4–9 mg/kg reported 7% cases of peripheral neuritis (Centre et al., 1963). Based on another pilot study, the TCC group proposed administering vitamin B complex formulation to prevent INH-induced neuritis (Centre, 1963). Table 2 provides a summary of various clinical trials highlighting peripheral neuropathy as a significant adverse effect. Altogether, these observations raised questions about the “safer dose” of INH. Also, if there is a need for simultaneous dosing of pyridoxine/vitamin B complex formulations with INH, what would be its suitable dose? Most of these clinical trials highlighted the favourable role of pyridoxine as a supplement in adults. An observational study in 1985 indicated a significant reduction in plasma pyridoxine levels in younger patients during INH treatment (Pellock et al., 1985). Based on the recommendations of the Joint Tuberculosis Committee, a new drug regimen consisting INH 10 mg/kg, RIF 10 mg/kg and pyridoxine 10 mg/kg was accepted for prophylactic use (Ormerod, 1987). This recommendation also led to a similar dose optimization study in INH Preventive therapy (IPT) for HIV-infected patients, as highlighted in Table 3. However, even after setting up a safer dose for INH and pyridoxine, still several clinical reports pointed out peripheral neuropathy as one of the adverse effects of isoniazid. The recent IMPAACT (International Maternal Pediatric Adolescent AIDS Clinical Trials) P1078 clinical trial reported 13% of neuritis cases by 24 weeks of treatment. The trial recruited 956 pregnant women under IPT to determine the prevalence of neurotoxicity in their *postpartum* period (Mandima et al., 2023). This also underlines two key points that need debate and further investigation. What is the safe and effective dose of pyridoxine? Is depletion of pyridoxine the sole factor responsible for neuritis? Chemically, the reactive–NH_2_ group of INH can form complex/adducts with many endogenous micro- and macromolecules, leading to the unavailability of these endogenous biomolecules. This becomes an important issue and a challenge, especially for IPT in pregnant women who are either diagnosed with HIV infection or latent TB infection. Additionally, the accumulation of INH in breast milk complicates this further. This leads to a pertinent question: is there a need to monitor pyridoxine levels in breastfed infants of infected mothers who are on INH treatment to study its impact on neurological development in infants? (Ng and Bandi, 2019). Importantly, CDC, American Thoracic Society (ATS) and Infectious Disease Society of America (IDSA) have already recommended the administration of pyridoxine as an adjunct along with INH at a dose of 1–2 mg/kg to breastfed infants given the probable risk of neurotoxicity among them (Nahid et al., 2016; Society, 2000).

**TABLE 2:** A summary of clinical trials and associated adverse events (focused on peripheral neuropathy) when INH was used as one of the drugs in treatment.

Country	Dose/Regimen	No. of volunteers	Adverse reactions/Side effects	Reference
United states	INH 20 mg/kg; PYX 100mg–450 mg	n = 20	Neuritis (n = 0)	Biehl and Vilter (1954a)
	INH 20 mg/kg; PYX 300mg–450 mg	n = 16	Neuritis (n = 14)	
Nigeria	INH 4 mg/kg – 6 mg/kg – daily – 6 to 12 monthsPAS 12 g – daily – 6–12 months	n = 84	Vasculo polyneuropathy (n = 16)	Money (1959)
United States	INH 20 mg/kg – daily – 20 weeks	n = 48	Peripheral neuropathy (n = 14)Convulsions (n = 1) Stupor, somnolence, mental confusion (n = 15)Dizziness, ataxia (n = 3)	Ross (1958)
	INH 20 mg/kg – daily – 20 weeksPYX 25 mg – twice daily – 20 weeks	n = 62	Peripheral neuropathy (n = 1)Convulsions (n = 2) Stupor, somnolence, mental confusion (n = 1)Dizziness, ataxia (n = 2)	
United states	INH 10 mg/kg – 16 mg/kg	n = 210	Peripheral neuropathy (n = 0)	Berte and Dewlett (1959)
	INH 10 mg/kg – 16 mg/kg; PYX 100 mg	n = 303	Peripheral neuropathy (n = 0)	
Hong Kong	INH 300mg; ETH 500 mg	n = 67	Peripheral neuropathy (n = 0)	Citron and Moodie (1964)
	INH 300mg; PAS 12 g	n = 69	Peripheral neuropathy (n = 0)	
Turkey	INH 5 mg/kg; RIF 10 mg/kg; PYR 25 mg/kg; ETA 15 mg/kg; STR 15 mg/kg – daily – 2 months INH 5 mg/kg; RIF 10 mg/kg – daily - 7 months	n = 1,149	Hepatotoxicity (n = 93) Hyperuricemia (n = 31) Ototoxicity (n = 19) Psychiatric changes (n = 8) Cutaneous reactions (n = 7) Flu-like syndrome (n = 3) Gluteal abscess (n = 2) Fever (n = 2) Peripheral neuropathy (n = 1) Visual acuity (n = 1) Haemolytic reactions (n = 1) Glucose tolerance (n = 1) Other side effects (n = 51)	Gülbay et al. (2006)
Uganda	RIF 150mg; INH 75mg; PYR 400mg; ETA 275 mg – daily – 2 months RIF 150mg; INH 75 mg – daily – 4 months	n = 268	Hepatotoxicity (n = 94) Peripheral neuropathy (n = 188)	Sekaggya-Wiltshire et al. (2017)

**TABLE 3:** A summary of clinical trials and adverse events associated with IPT.

Country	Dose	No. of volunteers	Adverse reactions/Side effects	Reference
United States	INH 300 mg	n = 2,321	Hepatic damage (n = 19)	Bagcchi (2023)
Unites States	INH 300 mg	n = 160	Adverse effects (n = 16)	Byrd et al. (1972)
Unites States	INH 300 mg	n = 1,000	Asymptomatic SGOT elevation (n = 47)Symptomatic SGOT elevation (n = 17)	Byrd et al. (1979)
South Africa	INH 300 mg	n = 338	Neuritis (n = 0)	Churchyard et al. (2003)
South Africa	INH 300 mg	n = 1,655	Peripheral neuropathy (n = 13)	Grant et al. (2005)
South Africa	INH 300mg; PYX 25 mg	n = 24,221	Peripheral neuropathy (n = 50), Hepatoxicity (n = 17), Hypersensitivity (n = 61), Convulsions (n = 4)	Grant et al. (2010)
Malawi	INH 300mg; PYX 25 mg	n = 869	Peripheral neuropathy (n = 19, n = 11), neutropenia (n = 31, n = 4), Anaemia (n = 30, n = 2), Gastro enteritis (n = 17, n = 10), Musculoskeletal pain (n = 10, n = 3), Leukopenia (n = 12, n = 0), Dizziness (n = 3, n = 8), Malaise (n = 6, n = 3), Rash (n = 3, n = 6)	Tsirizani-Galileya et al. (2022)
Australia	INH 300mg; PYX	n = 100	Peripheral neuropathy (n = 4)Gastrointestinal adverse effects (n = 21) Hepatitis (n = 5) Dermatological adverse effects (n = 15) Rashes (n = 15) Acne (n = 8) Neuropsychiatric adverse effects (n = 19) Lethargy (n = 7) Cognitive impairment (n = 9) Headache (n = 2) Sleep disturbance (n = 1) Depression (n = 1)	Denholm et al. (2014)
Korea	INH 300 mg	n = 114	Peripheral neuropathy (n = 6) Hepatotoxicity (n = 54)	Chung et al. (2020)

**Adverse effects reported as probably and possibly due to therapy.

In conclusion, the various clinical studies by multiple groups located in different geographical locations using different demography and drug regimens, highlighted the hepatotoxicity and neurotoxicity associated with the INH treatment. The *in vivo* metabolism of INH involving the–NH_2_ functional group is understood to be a significant cause for this. The issue of drug metabolism and its association with toxicity is discussed in the following section.

## 4 INH metabolism and its association with INH induced toxicity

**FIGURE 4:**
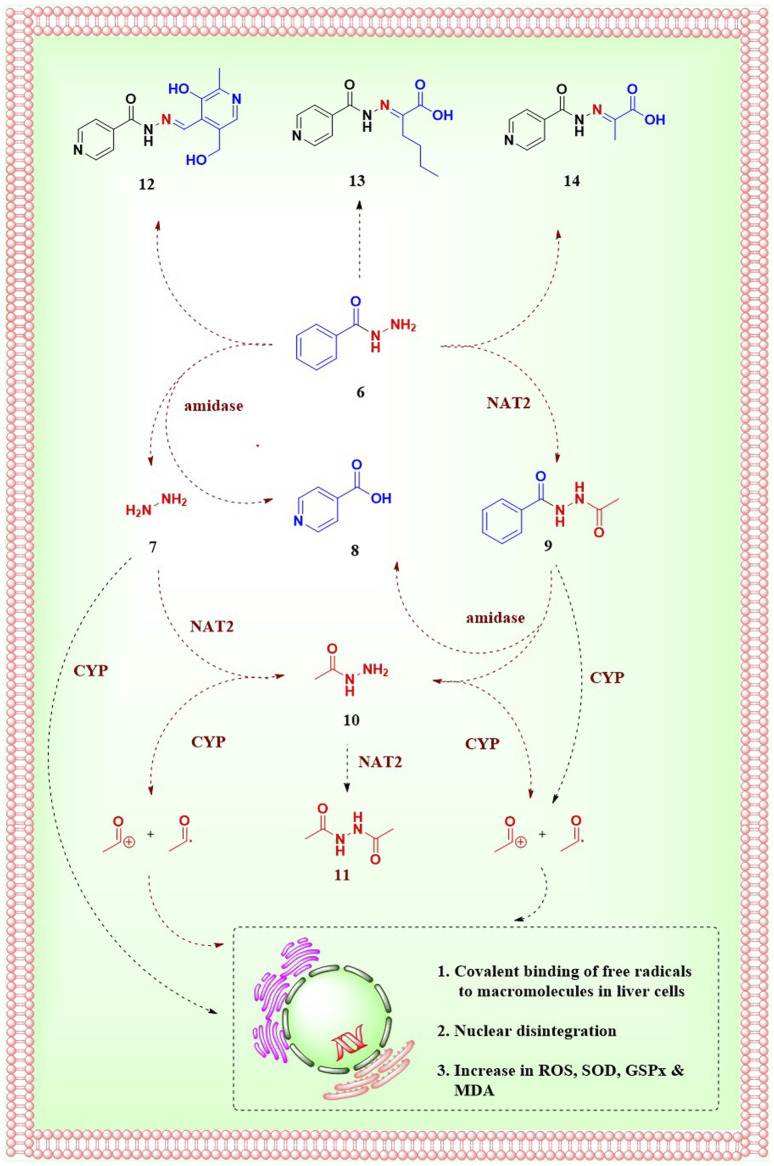
Metabolism of INH; 6.Isoniazid, 7.hydrazine, 8.Isonicotinic acid, 9.Acetylisoniazid, 10.Acetylhydrazine, 11.Diacetylhydrazine, 12.INH-pyridoxine hydrazone, 13.INH-α-ketoglutaric acid hydrazone, 14.INH–pyruvic acid hydrazone.

**FIGURE 5:**
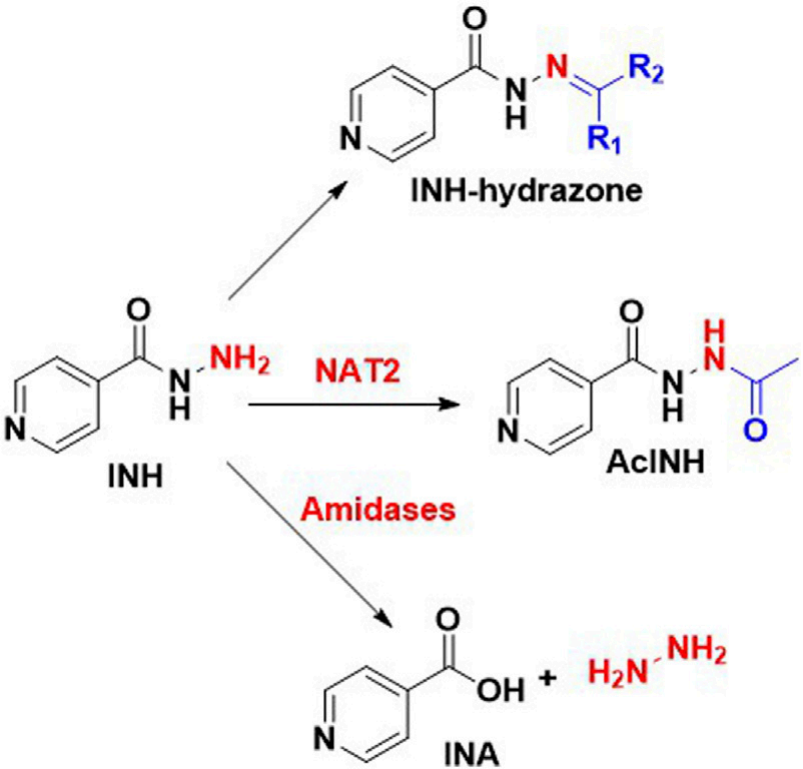
Major metabolic degradation pathways of INH in humans.

In general, the metabolism of a drug can happen in the gut, intestine, liver, plasma, or any tissue site. The liver is known to be the major site for INH metabolism. Within an hour of oral administration, a significant amount of free INH can be detected in the blood. INH is an amide analogue of isonicotinic acid (INA) having hydrazine (Hz) as an amine partner. The terminal and free -NH_2_ group of the hydrazide (-NH-NH_2_) in INH is chemically reactive being a good nucleophile. This higher reactivity of the terminal -NH_2_ group of INH is primarily responsible for its metabolism and covalent adduct formation with endogenous biomolecules. Post oral absorption, INH gets easily metabolised in hepatic cells leading to the generation of various metabolites like acetyl isoniazid (AcINH, 9; Figure 4), hydrazine (Hz, 7), acetyl hydrazine (AcHz, 10), diacetyl hydrazine (DiAcHz, 11), isonicotinic acid (INA, 8) by the action of enzymes like *arylamine N-acetyl transferase-2* (NAT-2) and amidases. Further, AcINH, AcHz and Hz are oxidised by P450 enzymes and form respective free radicals. These free radicals bind with hepatic macromolecules leading to toxicity and hepatic cell death. Additionally, INH itself is known to form covalent adducts (hydrazones) by interacting with various important endogenous biomolecules leading to toxicity (Mitchell et al., 1976; Preziosi, 2007; Erwin et al., 2019). Metabolism of INH transpires by two major pathways, and each involves different but overlapping sequences of chemical transformations (Figures 4, 5). The following section will elaborate on these metabolic transformations of INH and their consequences.

NAT-2, is a human enzyme also known as *arylamine N-acetyltransferases-2* and is predominantly expressed in the liver and gastrointestinal tract (Windmill et al., 2000). NAT-2 catalyses the acetylation of INH, which results in the production of AcINH. AcINH is an inactive form of INH. This is followed by hydrolysis of AcINH by host amidases, leading to the formation of INA and AcHz (Wang et al., 2016). Further, AcHz is either hydrolysed to Hz by amidases or can undergo another step of acetylation, leading to the formation of DiAcHz. Alternatively, INH itself gets hydrolysed by the action of amidases to release INA and Hz. The released Hz further gets acetylated to AcHz by the action of NAT-2 (Ellard and Gammon, 1976; McKennis et al., 1959). These metabolic degradation pathways of INH result in the formation of AcINH, INA, Hz, AcHz or DiAcHz as final metabolites as highlighted in Figure 4. Hydrazine and its derivative AcHz are notorious as hepatotoxic, carcinogenic and mutagenic agents (Metushi et al., 2011).

*Cytochrome P450* enzymes in the liver oxidise Hz and AcHz to form their corresponding free radicals inside the hepatic cells (Erwin et al., 2019). CYP2E1 is one of the major enzymes that is involved in INH metabolism (Delaney and Timbrell, 1995; Yue et al., 2004). Additionally, CYP2C and CYP2A are also known to catalyze this process. These free radical moieties and reactive intermediates (generated from different metabolites of INH) interact with endogenous macromolecules to form covalent adducts. These interactions and adducts of the INH metabolites are understood to be a major reason for INH-induced hepatotoxicity. The formation and interaction of these reactive intermediates with endogenous macromolecules have been studied extensively (Mitchell et al., 1976; Preziosi, 2007; Metushi et al., 2011; Nelson et al., 1976). Some of these free radicals induce a storm of reactive oxygen species beyond a threshold level, leading to an uncontrolled increase in oxidative stress inside hepatocytes. This has been confirmed by the unusual increase in superoxide dismutase (SOD), glutathione peroxidase (GSPx) and a decrease in malondialdehyde (MDA) in blood during INH administration (Sodhi et al., 1997; Sodhi et al., 1996; Huang et al., 2007). Similar to the oxidation of AcHZ or Hz, the P450 enzyme-mediated oxidation of INH forms reactive intermediates like diazohydroxide and diazene (Metushi et al., 2011; Metushi et al., 2012). These non-radical reactive species can potentially form adducts with endogenous micro and macromolecules. *In vitro* studies conducted by Meng et al. proved auto-oxidation of INH and subsequent formation of adducts with lysine residues in human serum albumin. The same adducts have also been detected in patients under INH prophylactic treatment (Metushi et al., 2016). Some of these adducts are anticipated to be potential source of immune-mediated idiosyncratic liver injury (Metushi et al., 2016; Uetrecht, 2009). The immune-mediated liver injury is supported by the detection of antiINH antibodies in patients who were under INH prophylactic treatment (Metushi et al., 2014). The possible mechanisms and clinical implications of INH-induced immune-mediated idiosyncratic liver injury have already been reviewed comprehensively (Metushi et al., 2016; Jee et al., 2021) and are beyond the scope of the topic as well as the intent of this article. Importantly, due to the issue of hepatotoxicity associated with INH treatment, sometimes TB therapy has to be withdrawn temporarily for a short period during the course of the treatment in vulnerable patients (Prasad et al., 2019).

In addition to these metabolic degradation pathways, INH itself reacts (non enzymatically) with endogenous carbonyl compounds like pyridoxine, α-keto glutaric acid and pyruvic acid to form corresponding hydrazones (12–14; Figure 4). As mentioned before, the higher nucleophilicity of the terminal -NH_2_ group of hydrazide in INH is responsible for the formation of these unwanted adducts. INH-Pyruvic acid hydrazone 14 is found to be more prominent than INH-keto glutaric acid hydrazone 13 (Zamboni and Defranceschi, 1954). Deficiency of vitamin B_6_ in the human body during INH treatment is attributed to these hydrazones formation. Vitamin B_6_ exists in 6 forms–pyridoxal, pyridoxamine, pyridoxine and their phosphorylated analogues. Among them, pyridoxal-5-phosphate is the major active form (Biehl and Vilter, 1954b). It is formed by the activation of pyridoxine by pyridoxine kinase enzyme. INH forms a stable hydrazone adduct 12 with pyridoxine, which gets excreted unchanged in the urine leading to the depletion of pyridoxal-5-phosphate in TB pateints (Wiegand, 1956; Mandel, 1959). As per the case reports of TB patients, the rate of excretion of vitamin B_6_ increases two fold of its normal rate of excretion (Glatstein et al., 2018). Limited consumption and uncontrolled excretion of pyridoxine with INH treatment results in deficiency of vitamin B_6_ (Mandel, 1959). In neurons, the glutamic acid decarboxylase enzyme synthesises γ-amino butyric acid (GABA) using pyridoxal-5-phosphate as a cofactor (Agarwal et al., 2016). The conditional deficiency of pyridoxal-5-phosphate due to INH treatment results in reduced GABA activation, leading to acute seizures in a few TB patients. In addition, INH itself inhibits the pyridoxine phosphokinase enzyme and restricts the phosphorylation of pyridoxine leading to deficiency of vitamin B_6_ (Minns et al., 2010). This gradual decrease in vitamin B6 and the development of seizures is a major cause of peripheral neuropathy. Reduced tendon reflexes, a feeling of numbness, paraesthesia, muscle pain, muscle weakness, and paralysis are typical characteristics of peripheral neuropathy.

The above-highlighted unwanted metabolism of INH is understood to be the reason for hepatotoxicity and peripheral neuropathy as two major adverse outcomes in the clinic. The liver injury or hepatotoxicity is clinically determined and diagnosed by an increase in the circulatory level of alanine aminotransferase (ALT) and alkaline phosphatase (ALP). Apart from clinical evidences, studies performed on rodents also highlighted the issue of hepatotoxicity associated with INH dosing. The studies conducted in mice and rabbits confirmed the toxic profile of AcHz and Hz. This study provided details on Hz-induced microvesicular steatosis and hepatic necrosis in rodent livers (Metushi et al., 2012; Sarich et al., 1996; Metushi and Uetrecht, 2014). Hz is reported to inhibit the functioning of mitochondrial complex - II and ATP production in hepatocytes leading to mitochondrial injury (Zentner et al., 2020).

The formation of INH metabolites and their plasma concentration is largely dependent on the activity and expression level of two host enzymes, i.e., NAT-2 and amidases. The altered level or expression of these enzymes can significantly alter the INH metabolism and hence toxicity associated with these metabolites or INH itself. For example, the expression of NAT-2 is not only tissue or gender-specific but also has interpersonal variability. NAT-2 is majorly expressed in the liver and gut. The acetylation rate of INH and the subsequent formation of various toxic and non-toxic metabolites depend on the NAT-2 expression and activity. Based on NAT-2 expression level and activity, human beings are phenotypically divided into two groups–slow acetylators and rapid acetylators. Slow acetylators have lower expression levels of NAT-2, resulting in restricted formation of toxic metabolites, and *vice versa* in rapid acetylators. Initially, it was assumed that rapid acetylators are at high risk of drug-induced liver injury (DILI) since toxic metabolites like AcINH and AcHz are rapidly formed (Mitchell et al., 1976; Mitchell et al., 1975; Yamamoto et al., 1986). Later on, a detailed understanding of NAT-2 polymorphisms revealed that treatment failure is more prominent in rapid acetylators, since the plasma concentration of INH is significantly insufficient due to its rapid metabolism leading to the formation of inactive AcINH (Parkin et al., 1997; Ellard, 1976; Donald et al., 2004). In contrast, hydrolysis of INH (formation of INA and Hz) is more dominant than acetylation (formation of AcINH, AcHZ or DiAcHz) in slow acetylators. This indirectly results in a higher accumulation of toxic metabolites like Hz and acetyldiazene. This observation was validated in the recently concluded clinical studies (Table 4), which imply that slow acetylators are significantly at high risk of DILI (Ohno et al., 2000; Huang et al., 2002). The meta-analysis of these studies reported recently in 2012 (Wang et al., 2012) and in 2019 (Khan S. et al., 2019) demonstrates the significant risk of hepatotoxicity in slow acetylators as compared to rapid acetylators. The data summarized in Table 3 highlights the outcome of similar global studies, establishing that the slow acetylators are more susceptible to DILI. There are reports which indicate that the severity and incidence of metabolism-associated toxicity of INH increase with the age of the patient (Oscanoa et al., 2023; Black et al., 1975; Bagcchi, 2023).

**TABLE 4:** Clinical outcomes of trials conducted to determine role of NAT-2 polymorphism in INH-DILI.

Year	Country	No. of patients	With Hepatotoxicity (%)	RA*/IA**-cases	RA/IA-Hepatotoxicity rate (%)	SA***-Cases	SA–Hepatoxicity rate	Reference
2002	Korea	132	18 (13.6%)	113	11 (9.7%)	19	7 (36.8%)	Cho et al. (2007)
2002	Taiwan	224	33 (14.7%)	171	19 (11.1%)	53	14 (26.4%)	Huang et al. (2002)
2011	India	218	41 (18.8%)	110	12 (10.9%)	108	29 (26.8%)	Bose et al. (2011)
2011	Japan	144	52 (36.1%)	131	44 (33.5%)	13	8 (61.5%)	Sotsuka et al. (2011)
2011	Iran	50	14 (28.0%)	36	5 (13.8%)	14	9 (64.3%)	Khalili et al. (2011)
2012	China	445	89 (20.0%)	353	71 (20.1%)	92	18 (19.5%)	Lv et al. (2012)
2012	China	208	101 (48.5%)	155	61 (39.3%)	53	40 (75.4%)	An et al. (2012)
2012	Tunisia	66	14 (21.2%)	33	3 (9.09%)	33	11 (33.3%)	Mahmoud et al. (2012)
2013	Brazil	270	18 (6.6%)	184	7 (3.8%)	86	11 (12.7%)	Santos et al. (2013)
2013	India	-	33	-	10	-	23	Mishra et al. (2013)
2013	India	215	50 (23.2%)	124	22 (17.7%)	91	28 (30.7%)	Gupta et al. (2013)
2014	China	2,244	89 (3.96%)	1,156	43 (3.71%	529	28 (5.29%)	Xiang et al. (2014)
2016	Indonesia	241	50 (20.7%)	144	18 (12.5%)	97	32 (32.9%)	Yuliwulandari et al. (2016)

*RA, Rapid Acetylators; **IA, Intermediate Acetylators; ***SA -Slow Acetylator.

## 5 Potential to improve pharmacological profile of INH

INH is inherently an active molecule prone to metabolism owing to high nucleophility of the terminal–NH_2_ group present in its structure. The metabolism of INH inside pathogens, i.e., bacteria, mediated by bacterial KatG enzyme leads to the activation of the INH, which is critical for antimicrobial potency of INH. However, undesired metabolism and reactivity of INH, while circulating inside the human host is a primary cause for drug-associated toxicity.

**FIGURE 6:**
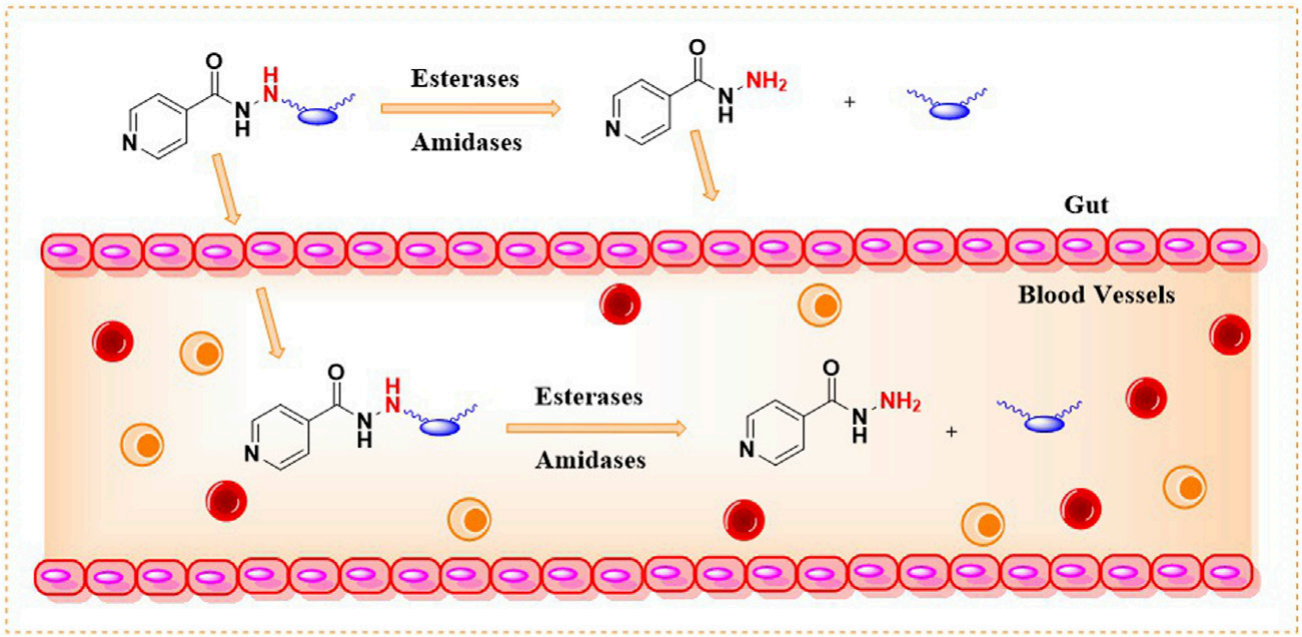
Controlled release of INH in gut and blood.

INH is an orally bioavailable drug that undergoes significant metabolism in the gut, liver, and plasma of the host, majorly via; 1) acetylation on the terminal -NH2 group of INH (N-acetylation) catalyzed by NAT-2 enzyme, 2) formation of isonicotinic acid due to hydrolysis of INH by amidases, 3) formation of hydrazones by reacting with vitamin B_6_ (Figure 5). The terminal –NH_2_ group of INH is prone to enzymatic (NAT-2 catalyzed) and non-enzymatic (e.g., hydrazone formation with pyridoxine, etc.) metabolic transformations. These metabolic transformations and their corresponding toxicological correlates has been nicely reviewed in past (Preziosi, 2007). The therapeutic dose monitoring of INH is proposed to be a possible solution for the NAT-2 based biotransformations, especially to address the issue of fast vs slow acetylators (Erwin et al., 2019). But real-time therapeutic dose monitoring has its own practical and logistic challenges, hence limited application. The non-enzymatic chemical transformation of INH (e.g., adduct formations) can be restricted by altering the chemical reactivity of the terminal –NH_2_ group. Additionally, the enzymatic biotransformation of any drug is understood to be very sensitive toward the three-dimensional structure of the drug due to the substrate structure specificity of the enzyme. It is also well understood that a slight change in the chemical structure of a drug not only changes the overall three-dimensional structure but also alters its electronic nature as well as physicochemical properties such as solubility, lipophilicity, and ionization potential. Together, these factors can modulate the drug’s absorption, distribution, metabolism, elimination, and pharmacokinetic (ADME-PK) profile in response to a small structural change. Chemically, it is possible to modify the structure of INH. There are successful reports in the literature where the *in vivo* metabolism issue of drugs (other than INH) has been addressed by chemically modifying them into labile derivatives or prodrugs (Subbaiah et al., 2024). Aripiprazole lauroxil is a recently approved drug by the FDA for schizophrenia that provides a sustained release and prolonged action of its active agent, aripiprazole (Najjar and Karaman, 2019). LY2334347 is an oral amide prodrug of gemcitabine, an anticancer agent that has currently completed phase I clinical trials (Yamamoto et al., 2013). Hence, it is logical to hypothesize that masking of the terminal –NH_2_ group by making a labile chemical derivative of INH, i.e., a derivative which can release INH back when exposed to biofluid in gut or blood (owing to hydrolytic activity of esterase/amidase) can limit known metabolic biotransformations of INH (Figure 6). A suitable labile structural alteration on the terminal –NH_2_ group of INH with low molecular weight moieties (having no or poor pharmacological implications) can potentially restrict its metabolism and related toxicities by enzyme-driven controlled release of INH in *in vivo* conditions.

Following the above-mentioned hypothesis, it is prudent to study two key research questions. First, can low molecular weight chemical derivatives of INH, such as amides and carbamates, undergo hydrolysis and release INH when exposed to biological fluids like whole blood or plasma due to the enzymatic action of esterases or amidases present in these biofluids? If so, the second logical research question would be: do these labile derivatives exhibit favorable plasma exposure and tissue distribution of INH when administered to animals and humans, owing to their different chemical structures and limited metabolism due to the masked terminal–NH_2_ group? Effectively, these new labile derivatives can alter the pharmacokinetic parameters like AUC, Cmax, Tmax, and T_1/2_ of INH compared to naïve INH. The prodrug approach has been well explored using amide and carbamate derivatives of various bioactive molecules (Subbaiah et al., 2024; Najjar and Karaman, 2019). Notably, the esterases and amidase-mediated *in vivo* drug release have been reported before for other drugs using similar derivatives (Yang et al., 2011). Chemically, it is possible to synthesise such new derivatives of INH (by exploiting the–NH_2_ group of INH) as potentially hydrolysable prodrugs of INH. Due to different chemical structures, the labile derivatives of INH will have different rates of absorption as well as release kinetics for INH *in vivo*, hence limiting the undesired metabolism of INH inside the host by enzymes such as NAT-2 or amidase. Additionally, a labile derivative with chemical masking of the terminal –NH_2_ group of INH can potentially restrict covalent adduct formation with endogenous macromolecules, unlike free INH. This chemical and, therefore, metabolic modulation of INH can potentially impact the bioavailability and metabolism-associated toxicity, improving the therapeutic index of INH leading to broader clinical applications. The argument for the possible toxicology profile of the modified INH as a new chemical entity will depend on the modified structure’s half-life and its release moieties. This concept of prodrug and labile masking has been exploited well in literature for drugs other than INH (Subbaiah et al., 2024; Najjar and Karaman, 2019).

A detailed literature search determined that the above-mentioned approach and corresponding research questions have not been exploited or studied for INH. However, a co-drug strategy exploiting conjugates of INH has been reported with the aim to ameliorate INH induced hepatotoxicity (Bhilare et al., 2018a; Bhilare et al., 2022). These two specific research reports (also discussed below) focused on the co-delivery of INH from a parent derivative, which is a covalently bonded conjugate of INH with another pharmacologically active agent possessing antioxidant properties. The aim of the conjugate is to ameliorate free radical stress induced by INH using the antioxidant properties of the other pharmacological agent. Unfortunately, the authors did not study the effect of the conjugation on the metabolism of the INH and did not discuss the effect of conjugation on the *in vivo* pharmacokinetic parameters of INH released from the parent derivative. Other than these, most of the reported studies around INH have been focused on creating new analogues or derivatives of INH aimed at better potency or efficacy without studying the effect of these derivatives on the release and/or metabolism of INH *in vivo*. Some very relevant reports focused on the synthesis and evaluation of INH derivatives, including conjugates, with an aim to restrict INH-induced toxicity, are summarised in the following section.

**FIGURE 7:**
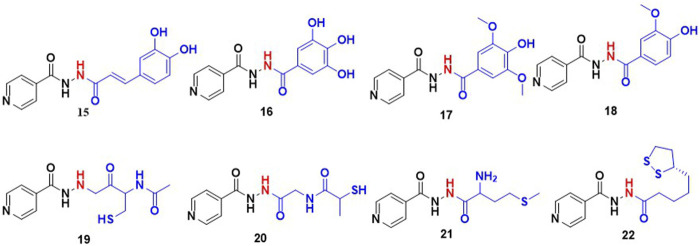
INH derivatives intended for lowering the oxidative stress.

**FIGURE 8:**
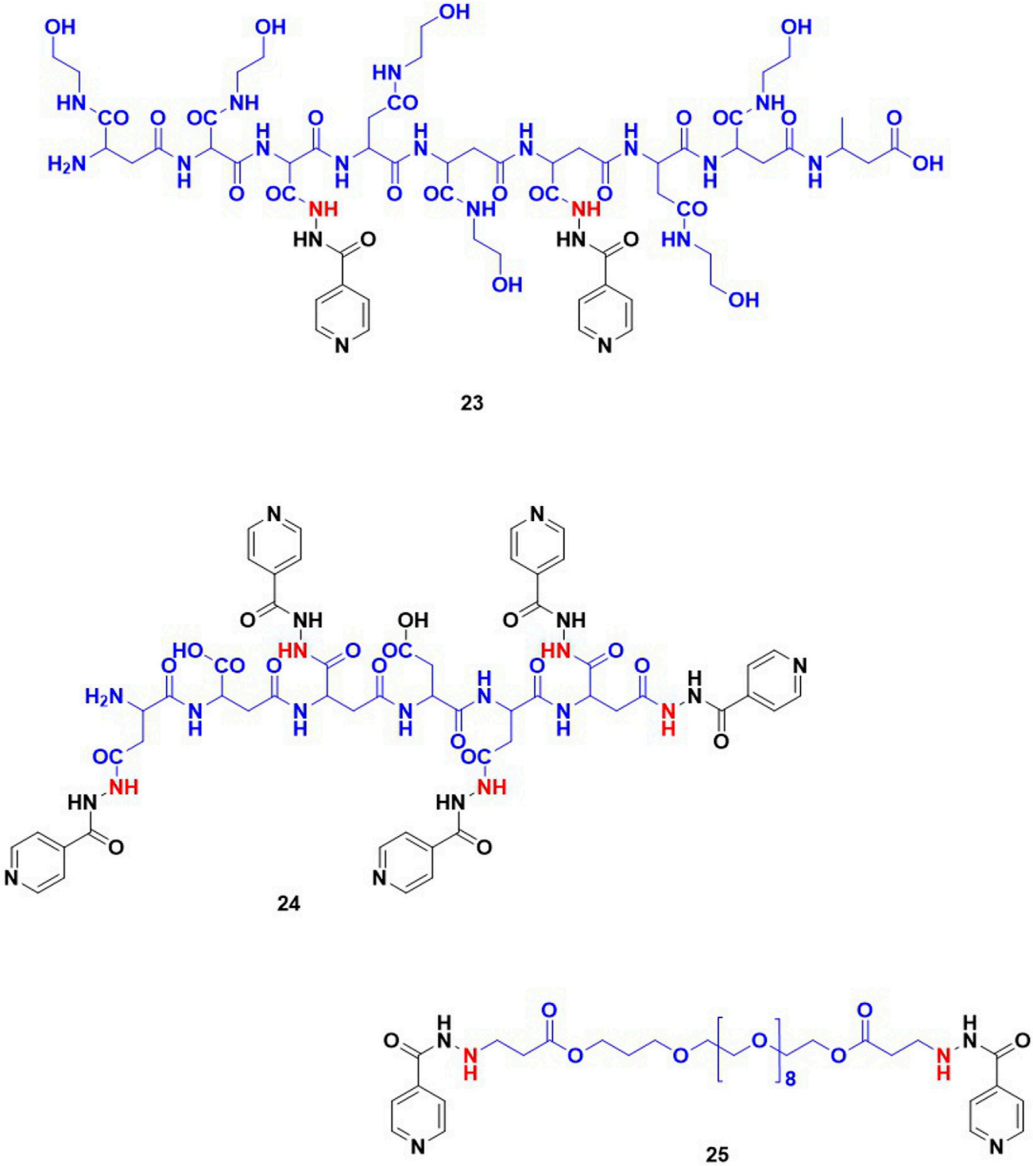
INH-poly aspartic acid/poly succinimide/poly ethylene glycol derivatives intended for controlled release of the compound.

Wu and co-workers studied the conjugates of INH with cinnamic acid and its derivatives. Cinnamic acid and its derivatives are reported to act as antioxidants because they can scavenge free radicals. This group evaluated the antimycobacterial activity and hepatoprotective potential of 10 bifunctional cinnamic acid hydrazide derivatives. MIC values of these new derivatives of INH varied in a range 10μM–100 μM with compound (15; Figure 7) reported as most potent with an MIC – 18.5 μM which is significantly poorer than the MIC of INH (0.36 μM). Histological analysis of liver tissues isolated from the mice group treated with compound 15 demonstrated an effective decrease in ALT, AST, MDA and a comparable increase in SOD, GSPx (Wu et al., 2015). It appeared that authors inferred and correlated the hepatoprotective effect to cinnamic acid. There is no data or comment either on *in vivo* release or metabolism of INH. Similar to cinnamic acids, some phenolic acids such as gallic acid, vanillic acid and syringic acid can also re-establish the antioxidant homeostasis in hepatocytes through their radical scavenging mechanisms (Badhani et al., 2015; Itoh et al., 2009). Hence the hepatoprotective profile and antitubercular activity of INH-phenolic acid conjugates were evaluated. Compounds 16–18, synthesized by the Schotten Baumann reaction as co-drug conjugates, were found to release INH by the action of intestinal homogenates as observed in *in-vitro* release studies. The *in-vivo* analysis revealed that the some of these compounds altered the levels of transaminase and antioxidant enzymes (Bhilare et al., 2018b). On similar lines, studies using aminothiols as anti-oxidant based conjugates of INH were also attempted. Aminothiols play a critical role in augmenting antioxidant machinery in a free radical rich environment. The derivatives of INH conjugated with N-acetylcysteine 19, L-methionine 20, and N- (2-mercapto propionyl) glycine 21 were also synthesized and evaluated with similar intent. Out of these three conjugates, 19 and 20 reported a higher release of INH (46% and 44% respectively) when incubated with stomach and intestinal homogenates. The histological analysis ranked these compounds in an order of 19 > 20 > 21 based on the favourable increase in antioxidant markers and a decrease in liver function markers (Bhilare et al., 2018a). The same research group has also explored the hepatoprotective potential of a sulphur containing compound, INH – α-lipoic acid combination 22, a co-drug strategy (Bhilare et al., 2022). Unfortunately, none of these reports mentioned data related to effect of these conjugates on known metabolic degradation of INH or *in vivo* pharmacokinetic parameters of INH. Additionally, there are reports focused on the controlled release formulations of INH. In most of these studies INH is conjugated with a typical slowly hydrolysable biopolymeric substances. Use of such formulations assured a prolonged existence of INH in blood. INH when covalently bonded with polyaspartic acid by an amide linkage resulted in 23 (Figure 8), is one among them. The research team behind 23 proposed that this could overcome the toxicity aroused by INH and might produce an improved biological activity. Using a similar approach, another prodrug is reported involving coupling INH with poly-succinimide i.e., compound 24. *In vitro* release kinetic studies determined a sustained release of INH from the 24 (Giammona et al., 1989). These studies inspired the synthesis and pharmacological evaluation of INH-ployethylene glycol (PEG) macromolecular conjugate, i.e., compound 25. PEG is an FDA approved drug carrier known for better biocompatibility and solubility (Kakkar et al., 2011; Kakkar et al., 2012; Cruz and Gil, 1998).

## 6 Concluding remarks

Therapeutic development against *Mtb* has been under intense research over the last 4 decades. On clinical fronts, efforts have been more focused on developing safer and shorter drug regimens by evaluating different permutations and combinations of existing approved drugs. On pre-clinical fronts, efforts are focused on the development of new or novel drug leads that can be progressed for first-in-human clinical study. In the recent past, there has been reasonably good progress and success on clinical fronts for the development of safer and shorter drug regimens using existing approved drugs against drug susceptibile as well as MDR and XDR TB infection. However, barring the recent discovery of bedaquilline and pretomanid, there has not been much success on the pre-clinical side in the last 40 years. It is unfair to compare clinical research with pre-clinical discovery efforts, especially in the case of TB infection. TB is not only a chronic infection demanding long treatment duration. Additionally, any newly identified drug lead has to pass the acid test of pre-clinical efficacy, safety, and compatibility with the existing first-line and second-line drugs. The pre-clinical efforts focused on the pharmacological improvement of the existing antitubercular drugs with a specific aim of reducing the therapeutic dose and increasing the therapeutic index by limiting *in vivo* metabolism is a less explored area. Research focused on abrogating the persisting drawbacks of existing drugs could provide an alternate, quicker and less expensive path to enhance treatment success for *Mtb* infection as well as broader use and application of existing drugs. INH is one of the oldest and most important first-line drugs for *Mtb* infection. Despite being a front-line drug, INH treatment is plagued with significant adverse effects. In addition, resistance to INH is another serious challenge in the clinic. These challenges in combination have led to repeated revisions of INH therapeutic regimens and combinations since its discovery in 1952. The treatment regimen of 6 months using INH led to serious adverse events like hepatotoxicity and peripheral neuropathy in some patients. This resulted in compliance issues, frequent dropouts or withdrawal of treatment (Scharer and Smith, 1969; Garibaldi et al., 1972). The primary cause of dose related toxicity of INH is associated with its *in vivo* metabolism. Biotransformation or metabolism of INH is majorly carried out by NAT-2 and amidases through acetylation and hydrolysis respectively. Biotransformation of INH inactivates INH and generates various inactive as well as toxic metabolites like AcINH, AcHz and Hz. These metabolites get oxidized into reactive free radicals inside the hepatocytes and are associated with hepatotoxicity. INH itself and its metabolites can potentially exert toxicity by forming hydrazones with various endogenous molecules. Most of these metabolic transformations involves the reactivity of the terminal–NH_2_ group of the INH. Herein, we propose and deliberate on a strategy to develop plasma labile derivatives of INH by chemical modification on the terminal -NH_2_ group to restrict undesired metabolism of INH. The hypothesis is that plasma labile INH derivatives or prodrugs exploiting terminal–NH_2_ of INH may restrict the host-directed metabolic transformations before its penetration inside the bacteria. The available clinical and preclinical literature on INH metabolism and its associated toxicities, combined with the success of the prodrug approach in other drugs, (Subbaiah et al., 2024; Najjar and Karaman, 2019) supported this hypothesis. There is a need to investigate this aspect systematically, as this approach could potentially restrict dose-related toxicity, leading to an improved therapeutic index of INH and, hence, better patient compliance and broader clinical use of the drug.

## Figures and Tables

**Table:** 

**INH**	Isoniazid
**RIF**	Rifampicin
**PZA**	Pyrazinamide
**ETA**	Ethambutol
**PAS**	Para amino salicylic acid
**STR**	Streptomycin
**THI**	Thioacetazone
**MDR**	Multidrug resistance
**XDR**	Extensive drug resistance
**DST**	Drug susceptibility test
**InhA**	NAPDH dependant enoyl acyl carrier protein reductase enzyme
**KasA**	β-Keto acyl ACP synthase I
**DHFR**	Dihydro folate reductase
**SGOT**	Serum glutamic oxalate acetic transaminase
**SGPT**	Serum glutamic pyruvic transaminase
**CDC**	Centre for disease control and prevention
**TCC**	Tuberculosis chemotherapy centre
**HIV**	Human immune deficiency virus
**GABA**	Gamma amino butyric acid
**ALT**	Alanine transaminase
**AST**	Aspartate aminotransferase
**ALP**	Alkaline phosphatase
**MDA**	Malondialdehyde
**SOD**	Superoxide dismutase
**GSPx**	Glutathione peroxidase
**MIC**	Minimum inhibitory concentration
**SAR**	Structure activity relationship
**PEG**	Polyethylene glycol
**FDA**	Food and drug administration
**PK**	Pharmacokinetics
**AUC**	Area under the curve.
