# Supplementary material for: Isoniazid-historical development, metabolism associated toxicity and a perspective on its pharmacological improvement
Source: Front Pharmacol. 2024 Sep 19;15:1441147. doi: 10.3389/fphar.2024.1441147 (PMC11447295; doi:10.3389/fphar.2024.1441147)
Supplement: Supplementary file 1 [file Table1.docx]

| **Supplementary Table 1.** A summary of clinical trials and associated adverse events(focused on hepatotoxicity) when INH was used as one of the drugs in treatment | | | | |
| --- | --- | --- | --- | --- |
| **Country** | **Dose/Regimen** | **No. of volunteers** | **Adverse reactions/ Side effects** | **Reference** |
| India | INH 200mg–daily-12 months | n=193 | Hepatistis (n=5) | ([136](#_ENREF_136)) |
|  | PAS 10g–daily–12 months |  |  |  |
| India | INH 200mg; PAS 10g–daily–one dose–12 months | n=71 | Hepatitis (n=2) | ([137](#_ENREF_137)) |
|  | INH 300mg; THI 150mg–daily–one dose–12 months | n=75 | Hepatitis (n=2) |  |
|  |  |  | Exfoliative dermatistis, (n=3) |  |
|  | INH 200mg; PAS 6g–daily–one dose–6 months | n=71 | Hepatitis (n=0) |  |
|  | INH 300mg–daily–one dose–6 months |  |  |  |
| International Study | INH 300mg; THI 150mg; STR 1g–daily–one dose-8 weeks | n=1002 | Hepatitis (n=2) | ([138](#_ENREF_138)) |
|  |  |  | Nausea (n=40) |  |
|  |  |  | Vomiting (n=43) |  |
|  |  |  | Flushing/Itching (n=13) |  |
|  |  |  | Skin rashes (n=39) |  |
|  |  |  | Dizziness (n=96) |  |
|  |  |  | Vertigo (n=9) |  |
|  |  |  | Ataxia (n=14) |  |
|  |  |  | Tinnitus (n=4) |  |
|  |  |  | Deafness (n=7) |  |
|  |  |  | Death(n=2)* |  |
|  | INH 300mg; STR 1g–daily–one dose-8 weeks | n=987 | Hepatitis (n=3) |  |
|  |  |  | Nausea (n=16) |  |
|  |  |  | Vomiting (n=5) |  |
|  |  |  | Flushing/Itching (n=5) |  |
|  |  |  | Skin rashes (n=10) |  |
|  |  |  | Dizziness (n=29) |  |
|  |  |  | Vertigo (n=2) |  |
|  |  |  | Ataxia (n=5) |  |
|  |  |  | Death (n=1)* |  |
| India | STR 0.75g–1g; INH 750mg–weekly–two doses–12 months | n=104 | Jaundice (n=0) | ([139](#_ENREF_139)) |
|  |  |  |  |  |
|  | STR 0.75g–1g; INH 750mg–weekly–one dose–12 months | n=79 | Jaundice (n=1) |  |
|  |  |  |  |  |
|  | STR 0.75g–1g; INH 750mg; PZA 4g–weekly–one dose | n=105 | Jaundice (n=2) |  |
|  | –12 months |  |  |  |
|  | STR 0.75g–1g; INH 400mg–daily–one dose–4 weeks | n=106 | Jaundice (n=0) |  |
|  | STR 0.75g–1g; INH 750mg–weekly–one dose–48 weeks |  |  |  |
| Singapore | INH 300mg; TH 150mg; STR 1g–daily–one dose–6 months | n=128 | Hepatitis (n=4) | ([140](#_ENREF_140)) |
|  | INH 300mg; THI 150mg–daily–one dose–6 months |  |  |  |
|  | INH 300mg; TH 150mg–daily–one dose–12 months | n=100 | Hepatitis (n=4) |  |
|  |  |  | Death (n=1)* |  |
|  | INH 300mg; STR:1g–daily–one dose–6 months | n=131 | Hepatitis (n=2) |  |
|  | INH 300mg–daily–one dose–6 months |  |  |  |
| Scotland | INH 300mg; RIF 300mg–3 months | n=105 | Hepatitis (n=14) | ([141](#_ENREF_141)) |
|  |  |  | Jaundice (n=1) |  |
|  |  |  | Skin rashes (n=2) |  |
| International Study | INH 300mg; THI 150mg; STR 1g–daily–one dose | n=1396 | Nausea (n=165) | ([142](#_ENREF_142)) |
|  |  |  | Vomiting (n=143) |  |
|  |  |  | Diarrhoea (n=12) |  |
|  |  |  | Constipation (n=43) |  |
|  |  |  | Hepatitis (n=5) |  |
|  |  |  | Flushing/Itching (n=90) |  |
|  | – 12 weeks |  | Skin rashes (n=254) |  |
|  |  |  | Dizziness (n=316) |  |
|  |  |  | Ataxia (n=142) |  |
|  |  |  | Tinnitus (n=16) |  |
|  |  |  | Death (n=7) |  |
|  | INH 300mg; STR 1g–daily–one dose–12weeks | n=1407 | Nausea (n=77) |  |
|  |  |  | Vomiting (n=35) |  |
|  |  |  | Diarrhoea (n=22) |  |
|  |  |  | Constipation (n=21) |  |
|  |  |  | Hepatitis (n=2) |  |
|  |  |  | Flushing/Itching (n=60) |  |
|  |  |  | Skin rashes (n=114) |  |
|  |  |  | Dizziness (n=165) |  |
|  |  |  | Ataxia (n=57) |  |
|  |  |  | Tinnitus (n=10) |  |
|  |  |  | Death (n=9) |  |
|  | INH 300mg; THI 150mg–daily–one dose–12 weeks | n=1165 | Gastric disturbances (n=188) |  |
|  |  |  | Cutaneous disturbances (n=253) |  |
|  |  |  | Vestibular (n=86) |  |
| Britain | INH 300mg; RIF 450mg; STR 0.75mg–daily–3 months | n=63 | Hepatitis (n=4) | ([143](#_ENREF_143)) |
| India | STR 1g; PAS 6g; INH 400mg; PZA 6g–First two weeks | n=217 | Jaundice (n=2) | ([144](#_ENREF_144)) |
|  | PAS 0.2g/Kg; INH 15mg/Kg; PZA 6mg–weekly–two doses | n=109 | Jaundice (n=4) |  |
|  |  |  |  |  |
|  | PAS–0.2g/Kg; INH 4.7mg/Kg–daily–one dose | n=108 | Death (n=1) |  |
| India | STR 0.75g–1g–daily–one dose–4 weeks | n=181 | Jaundice (n=5) | ([145](#_ENREF_145)) |
|  | INH 13mg/kg–weekly–one dose–4 weeks |  |  |  |
|  | STR 0.75g–1g; INH 17mg/kg–weekly–one dose–48 weeks |  |  |  |
|  |  |  |  |  |
|  | STR 0.75–1g; INH 400mg; PAS 6g–daily–one dose–4 weeks | n=178 | Jaundice (n=3) |  |
|  |  |  |  |  |
|  | STR 0.75g–1g; INH 15mg/kg; PAS 6g–weekly–one dose– 48 weeks |  |  |  |
|  |  |  |  |  |
| India | STR 1g; INH 400mg; PAS 6g–daily–one dose–2 weeks | n=111 | Jaundice (n=2) | ([144](#_ENREF_144)) |
|  | INH 15mg/kg; PAS 0.2g/kg–weekly–two doses–50 weeks |  |  |  |
|  |  |  |  |  |
|  | STR–1g; INH 400mg; PAS 6g–daily–one dose–2 weeks | n=109 | Jaundice (n=4) |  |
|  |  |  |  |  |
|  | INH 5mg/kg; PAS 0.2g/kg–daily–one dose–50 weeks |  |  |  |
| India | STR 0.75g; INH 400mg–daily–one dose–4 weeks | n=81 | Jaundice (n=2) | ([145](#_ENREF_145)) |
|  | STR 0.75g; INH 15mg/kg–weekly–one dose–48 weeks |  |  |  |
|  | STR 0.75g–1g–daily–one dose–4 weeks | n=173 | Jaundice (n=3) |  |
|  | INH 30mg/kg–weekly–one dose–4 weeks |  |  |  |
|  | STR 0.75g–1g; INH 30mg/kg–weekly–one dose–48 weeks |  |  |  |
|  | STR 0.75g–1g daily–one dose–4 weeks | n=142 | Jaundice (n=0) |  |
|  | INH 40mg/kg–weekly–one dose–4 weeks |  |  |  |
|  | STR 0.75g–1g;INH 40mg/kg–weekly–one dose–48 weeks |  |  |  |
| United States | INH 300mg-daily-12 months | n=13838 | Hepatitis (n=92, n=82)* | ([146](#_ENREF_146)) |
| Netherlands | INH 300mg; RIF 600mg; ETA 25mg/Kg–daily–6 months | n=103 | Hepatotoxicity (n=10) | ([147](#_ENREF_147)) |
|  | INH 300mg; RIF 600mg; ETA 25mg/Kg–daily–one dose | n=105 | Hepatotoxicity (n=11) |  |
|  | –2 months |  | Thrombocytopaenia (n=4) |  |
|  | INH 300mg; RIF 600mg–weekly–two doses 4 months |  |  |  |
|  | INH 300mg; RIF 600mg; ETA 25mg/Kg–daily–one dose | n=102 | Hepatotoxicity (n=7) |  |
|  | –2 months |  | Thrombocytopaenia (n=2) |  |
|  | INH 300mg; RIF 600mg; ETA 25mg/Kg–weekly–one dose |  | Gastrointestinal disturbance (n=2) |  |
|  | –4 months |  |  |  |
|  | INH 300mg; RIF 600mg; ETA 25mg/Kg–daily–two doses | n=101 | Hepatotoxicity (n=7) |  |
|  | -4 months |  | Thrombocytopaenia (n=1) |  |
|  | INH 300mg; RIF 600mg; ETA 25mg/Kg–weekly-two doses |  | Gastrointestinal disturbance(n=1) |  |
|  | –4 months |  |  |  |
|  | INH 300mg; RIF 600mg; PZA 2g; STR 1g-daily– | n=119 | Hepatotoxicity (n=13) |  |
|  | one dose–2 months |  | Thrombocytopaenia (n=1) |  |
|  | INH 300mg; RIF 600mg-weekly–2 doses–4 months |  | Gastrointestinal disturbances(n=1) |  |
| Britain | INH 300mg; RIF 600mg-daily–6 months | n=170 | Hepatitis (n=21) | ([148](#_ENREF_148)) |
|  | STR 0.75g ; PZA 1.5g–daily-2 months |  |  |  |
|  | INH 300mg; RIF 600mg-daily–6 months | n=164 | Jaundice (n=8) |  |
|  | ETA 25mg/Kg; PZA 1.5g–daily–2 months |  |  |  |
|  | INH 300mg; RIF 600mg–daily–9 months | n=177 | Gastrointestinal disturbances |  |
|  | ETA 25mg/Kg–daily–2 months |  | (n=10) |  |
| China | INH 15mg/kg; RIF 600mg; STR 1g; PZA 2g or 2.5g; | n=244 | Gastrointestinal disturbances (n=57) | ([149](#_ENREF_149)) |
|  | ETA 30mg/Kg–weekly–3 doses–6 months |  | Hepatotoxicity (n=3) |  |
|  | INH 15mg/kg; RIF 600mg; STR 1g; | n=243 | Gastrointestinal disturbances (n=40) |  |
|  | ETA 30mg/Kg–weekly–3 doses–6 months |  | Hepatotoxicity (n=2) |  |
|  | INH 15mg/kg; RIF 600mg; STR 1g; | n=239 | Gastrointestinal disturbances (n=20) |  |
|  | PZA 2g or 2.5g–weekly–3 doses–6 months |  | Hepatotoxicity (n=0) |  |
|  | INH 15mg/kg; RIF 600mg; PZA 2g or 2.5g; | n=242 | Gastrointestinal disturbances (n=42) |  |
|  | ETA 30mg/Kg–weekly–3 doses–6 months |  | Hepatotoxicity (n=3) |  |
|  | INH 300mg; RIF 450mg or 600mg; PZA 1.5g or 2g; | n=239 | Gastrointestinal disturbances (n=21) |  |
|  | ETA 25mg/Kg–weekly–3 doses–6 months |  | Hepatotoxicity (n=13) |  |
| India | STR 1g; INH 400mg; ETA 25mg/kg–daily–one dose–2 weeks | n=107 | Jaundice (n=2) | ([150](#_ENREF_150)) |
|  | INH 400mg; ETA 15mg/kg–daily–one dose–50 weeks |  |  |  |
|  | STR 1g; INH 400mg; ETA 25mg/kg–daily–one dose–2 weeks | n=101 | Jaundice (n=2) |  |
|  | INH 15mg/kg; ETA 45mg/kg–weekly–two doses–50 weeks |  |  |  |
|  | STR 1g;INH 400mg;ETA 25mg/kg–daily–one dose–two weeks | n=107 | Jaundice (n=1) |  |
|  | INH 15mg/kg–weekly–two doses–50 weeks |  |  |  |
|  | ETA 90mg/kg–weekly–one dose–50 weeks |  |  |  |
|  | STR 1g; INH 400mg; ETA 25mg/kg-daily-one dose-two weeks | n=109 | Jaundice (n=1) |  |
|  | INH 15mg/kg; ETA 90mg/kg–weekly–one dose–50 weeks |  |  |  |
| India | STR 0.75g; INH 400mg; RIF 12mg/kg; PZA 40mg/kg–daily– | n=170 | Jaundice (n=5) | ([151](#_ENREF_151)) |
|  | one dose–2 months |  |  |  |
|  | STR 0.75g; INH 15mg/kg; PZA 70mg/kg–weekly–two doses |  |  |  |
|  | –3 months |  |  |  |
|  | STR 0.75g; INH 400mg; RIF 12mg/kg; PZA 40mg/kg–daily– | n=168 | Jaundice (n=7) |  |
|  | one dose–2 months |  |  |  |
|  | STR 0.75g; INH 15mg/kg; PZA 70mg/kg–weekly–two doses |  |  |  |
|  | –5 months |  |  |  |
|  | STR 0.75g; INH 400mg; PZA 40mg/kg-daily-one dose-2 months | n=345 | Jaundice (n=4) |  |
|  | STR 0.75g; INH 15mg/kg; PZA 70mg/kg–weekly–two doses |  |  |  |
|  | –5 months |  |  |  |
| United States | INH 300mg; RIF 600mg–daily–one dose–6 months | n=309 | Hepatotoxicity (n=37) | ([152](#_ENREF_152)) |
|  | INH 300mg; ETA 15mg/Kg–daily–one dose–9 months |  |  |  |
| United States | INH 15-20mg/kg; RIF 15mg/kg | n=44 | Hepatitis and Jaundice (n=15) Liver enlargement (n=7) | ([153](#_ENREF_153)) |
| Pakistan | RIF 450mg; INH 300mg; PZA 1.5g or 2g–daily–one dose | n=118 | Side effects (n=1) | ([154](#_ENREF_154)) |
|  | –6 months |  |  |  |
|  | RIF 450mg; INH 300mg–daily–one dose–6 months | n=117 | Adverse effect (n=1) |  |
|  | ETA 25mg/kg–daily–one dose–2 months |  |  |  |
|  | ETA 15mg/kg–daily–one dose–4 months |  |  |  |
|  | STR 1g; INH 300mg–daily–one dose–6 months | n=117 | Adverse effect (n=0) |  |
|  | ETA 25mg/kg–daily–one dose–2 months |  |  |  |
|  | ETA 15mg/Kg–daily–one dose–4 months |  |  |  |
| India | RIF 12mg/kg; STR 0.75g; INH 400mg; PZA 35mg/kg;-daily | n=173 | Hepatitis (n=16) | ([155](#_ENREF_155)) |
|  | –one dose–3 months |  |  |  |
|  | RIF 12mg/kg; STR 0.75g; INH 400mg; PZA 35mg/kg-daily– | n=180 | Hepatitis (n=16) |  |
|  | one dose–3 months |  |  |  |
|  | STR 0.75g; INH 15mg/kg; PZA 70mg/kg–weekly–two doses– |  |  |  |
|  | two months |  |  |  |
|  | STR 0.75g; INH 400mg; PZA 35mg/kg-daily–one dose | n=179 | Hepatitis (n=1) |  |
|  | –3 months |  |  |  |
|  | STR 0.75g; INH 15mg/kg; PZA 70mg/kg–weekly–two doses– |  |  |  |
|  | two months |  |  |  |
| United States | INH 3-5mg/Kg; PAS 8-12g–daily–24 weeks | n=123 | Hepatitis (n=1) | ([156](#_ENREF_156)) |
|  | INH 3-5mg/Kg; PZA 25mg/Kg–daily–24 weeks | n=160 | Hepatitis (n=4) Jaundice (n=1) |  |
|  | INH 3-5mg/kg; PZA 40mg/Kg–daily–24 weeks | n=167 | Hepatitis (n=11) |  |
|  |  |  | Jaundice (n=4) |  |
| United States | INH 1.2g; PZA 100mg–daily–1 month | n=11 | Hepatoxicity (n=1) | ([156](#_ENREF_156)) |
|  |  |  |  |  |
| Brazil | INH 400mg; RIF 600mg; PYR 2g–daily–2 months INH 400mg; RIF 600mg–daily–4 months | n=287 | Death (n=2) Adverse effects (n=2) | ([157](#_ENREF_157)) |
|  | INH 400mg; RIF 600mg; PYR 2g–daily–2 months INH 400mg; RIF 600mg–twice weekly–4 months | n=257 | Adverse effects (n=3) |  |
| United States, Canada, Brazil, Hong Kong, Spain | INH 5mg/Kg(>12yrs), 10mg/Kg(2–11 yrs)–daily–9 months | n=506 | Adverse effect (n=6) | ([158](#_ENREF_158)) |
|  | RIF 300mg–900mg–weekly–3 months INH 15mg/Kg (>12yrs), 25mg/Kg (2–11 yrs)–weekly–3 months | n=552 | Adverse effect (n=14) |  |
| Australia, Benin, Brazil, Canada, Ghana, Guinea, Indonesia, Audi Arabia, South Korea | INH 5mg/Kg–daily–9 months | n=2809 | Hepatotoxicity (n=140)  Death (n=5) | ([159](#_ENREF_159)) |
|  | RIF–10mg/Kg–daily–9 months | n=2887 | Hepatotoxicity (n=24) |  |
| Australia, Benin, Brazil, Canada, Ghana, Guinea, Indonesia | INH 10–15 mg/Kg–daily–9 months | n=407 | Adverse effect (n=0) | ([160](#_ENREF_160)) |
|  | RIF 10–20 mg/Kg–daily–9 months | n=422 | Adverse effect (n=0) |  |
| *Adverse effects reported as probably and possibly due to therapy. | | | | |

**References of information in Table S1:**

136. Centre TC. A concurrent comparison of home and sanatorium treatment of pulmonary tuberculosis in South India. Bulletin of the World Health Organization. 1959;21(1):51.

137. Centre TC. Isoniazid plus thioacetazone compared with two regimens of isoniazid plus PAS in the domiciliary treatment of pulmonary tuberculosis in South Indian patients. Bulletin of the World Health Organization. 1966;34(4):483.

138. Miller A, Fox W, Tall R. An international co-operative investigation into thiacetazone (thioacetazone) side-effects. Tubercle. 1966;47(1):33-74.

139. Centre TC. A controlled comparison of a twice-weekly and three once-weekly regimens in the initial treatment of pulmonary tuberculosis. Bulletin of the World Health Organization. 1970;43(1):143.

140. Services ST, Hospital B. A controlled clinical trial of the role of thiacetazone-containing regimens in the treatment of pulmonary tuberculosis in Singapore. Tubercle. 1971;52(2):88-116.

141. Lees A, Allan G, Smith J, Tyrrell W, Fallon R. Toxicity from rifampicin plus isoniazid and rifampicin plus ethambutol therapy. Tubercle. 1971;52(3):182-90.

142. Miller A, Nunn A, Robinson D, Fox W, Somasundaram P, Tall R. A second international cooperative investigation into thioacetazone side effects: 2. Frequency and geographical distribution of side effects. Bulletin of the World Health Organization. 1972;47(2):211.

143. Lal S, Singhal S, Burley D, Crossley G. Effect of rifampicin and isoniazid on liver function. British Medical Journal. 1972;1(5793):148-50.

144. OI A. Controlled comparison of oral twice-weekly and oral daily isoniazid plus PAS in newly diagnosed pulmonary tuberculosis. British Medical Journal. 1973;2:7-11.

145. Tripathy S. A slow-release preparation of isoniazid: Therapeutic efficacy and adverse side-effects. Bulletin of the International Union Against Tuberculosis. 1976;51(1):133-41.

146. Kopanoff DE, Snider Jr DE, Caras GJ. Isoniazid-related hepatitis: a US Public Health Service cooperative surveillance study. American review of respiratory disease. 1978;117(6):991-1001.

147. Zierski M, Bek E. Side-effects of drug regimens used in short-course chemotherapy for pulmonary tuberculosis. A controlled clinical study. Tubercle. 1980;61(1):41-9.

148. Somner A, Association BT. A controlled trial of six months chemotherapy in pulmonary tuberculosis: first report: results during chemotherapy. British Journal of Diseases of the Chest. 1981;75(2):141-53.

149. Service HKC, Council BMR. Controlled trial of four thrice-weekly regimens and a daily regimen all given for 6 months for pulmonary tuberculosis. The Lancet. 1981;317(8213):171-4.

150. Centre TR. Ethambutol plus isoniazid for the treatment of pulmonary tuberculosis-A controlled trial of four regimens. Tubercle. 1981;62(1):13-29.

151. Centre TR. Study of chemotherapy regimens of 5 and 7 months' duration and the role of corticosteroids in the treatment of sputum-positive patients with pulmonary tuberculosis in South India. Tubercle. 1983;64(2):73-91.

152. Snider Jr D, Long M, Cross F, Farer L. Six-months isoniazid-rifampin therapy for pulmonary tuberculosis. Report of a United States Public Health Service Cooperative Trial. The American review of respiratory disease. 1984;129(4):573-9.

153. Tsagaropoulou-stinga H, Mataki-emmanouilidou T, Karida-kavalioti S, Manios S. Hepatotoxic reactions in children with severe tuberculosis treated with isoniazid-rifampin. The Pediatric Infectious Disease Journal. 1985;4(3):270-3.

154. Aziz A, Ishaq M, Jaffer N, Akhwand R, Bhatti A. Clinical Trial of Two Short-course (6-Month) Regimens and a Standard Regimen (12-Month) Chemotherapy in Retreatment of Pulmonary Tuberculosis in Pakistan: Results 18 Months after Completion of Treatment (Lahore Tuberculosis Study). American Review of Respiratory Disease. 1986;134(5):1056-61.

155. Parthasarathy R, Sarma GR, Janardhanam B, Ramachandran P, Santha T, Sivasubramanian S, et al. Hepatic toxicity in South Indian patients during treatment of tuberculosis with short-course regimens containing isoniazid, rifampicin and pyrazinamide. Tubercle. 1986;67(2):99-108.

156. Hallett M, Ravits J, Dubinsky RM, Gillespie MM, Moinfar A. A double‐blind trial of isoniazid for essential tremor and other action tremors. Movement Disorders: Official Journal of the Movement Disorder Society. 1991;6(3):253-6.

157. Castelo A, Goihman S, Dalboni M, Jardim JB, Kalckman A, Da Silva E, et al. Comparison of daily and twice-weekly regimens to treat pulmonary tuberculosis. The Lancet. 1989;334(8673):1173-6.

158. Villarino ME, Scott NA, Weis SE, Weiner M, Conde MB, Jones B, et al. Treatment for preventing tuberculosis in children and adolescents: a randomized clinical trial of a 3-month, 12-dose regimen of a combination of rifapentine and isoniazid. JAMA pediatrics. 2015;169(3):247-55.

159. Menzies D, Adjobimey M, Ruslami R, Trajman A, Sow O, Kim H, et al. Four months of rifampin or nine months of isoniazid for latent tuberculosis in adults. New England Journal of Medicine. 2018;379(5):440-53.

160. Diallo T, Adjobimey M, Ruslami R, Trajman A, Sow O, Obeng Baah J, et al. Safety and side effects of rifampin versus isoniazid in children. New England Journal of Medicine. 2018;379(5):454-63.
